# Fabrication of Rectification Nanosensors by Direct Current Dielectrophoresis Alignment of ZnO Nanowires

**DOI:** 10.1186/s11671-021-03539-6

**Published:** 2021-05-19

**Authors:** Kai-Heng Sun, Wen-Ching Chien, Hsun-Feng Hsu

**Affiliations:** Department of Materials Science and Engineering, National Chung Hsing Univiersity, 145 Xingda Rd., Taichung, 40227 Taiwan

**Keywords:** ZnO, Nanowire, Dielectrophoresis, Photodetector, Rectifying device

## Abstract

This work demonstrates the fabrication and characterization of ZnO nanowire-based devices in a metal–nanowire–metal configuration using the direct current dielectrophoresis alignment across Au electrodes. The current–voltage characteristics of the devices revealed that they were rectifying, and the direction of rectification was determined by the direction of current due to the asymmetric Joule heating in the dielectrophoresis alignment process. Joule heating caused the Au atoms to diffuse from the Au electrodes to the inner ZnO NWs and the formation of Schottky contact at the Au/ZnO interface. A fast and sensitive photoresponse was achieved for the rectifying devices in reverse-biased mode due to the carrier injection and photocurrent gain under UV illumination. Such direct current dielectrophoresis alignment of ZnO nanowires is a facile method for fabricating rectification devices with application in sensitive and fast UV detecting sensors.

**Supplementary Information:**

The online version contains supplementary material available at 10.1186/s11671-021-03539-6.

## Introduction

ZnO is an n-type metal-oxide semiconductor with a direct band gap energy of 3.37 eV. Recently, ZnO nanowires (NWs) have attracted interest owing to their potential use in ultraviolet (UV) sensors [1] because of their high surface-to-volume ratio, high electron–hole generation and recombination rates, high conductivity, and non-toxicity. Various methods, such as vapor phase transport processes [2], chemical vapor deposition (CVD) [3], and hydrothermal methods [4, 5], have been used to synthesize ZnO NWs. Among these techniques, the hydrothermal method is the most cost-effective for mass production.

In recent years, research for fabrication of high-performance UV sensors based on ZnO NWs has been reported [6–10]. In which, the significant reduction of dark current could improve the sensitivity of sensors. The rectifying I–V characteristics of devices that include both a pn junction and Schottky contact diodes could achieve this purpose [11–16]. The use of a Schottky diode can not only improve the sensitivity of the devices but can also reduce the response time. However, in previous studies, the fabrication of nanowire Schottky diodes was very complicated. For example, one end of ZnO nanowire is brought into contact with a Cu or Pt electrode by dielectrophoretic deposition, placing or e-beam lithography methods are used to make a Schottky contact, and the other end is formed an Ohmic contact through the deposition of Pt/Ga using FIB. [11–13]

Dielectrophoresis (DEP) is one of the methods that is commonly used to align NWs in the fabrication of sensors in metal–semiconductor–metal structures because it is a simple, low-cost method and can be used not only for single NW alignment but also for the large-area alignment of multi-segmented NWs. Dielectric NWs can align precisely across electrodes when NWs are subjected to a non-uniform electric filed that is generated by an alternating current (AC). The devices with rectifying I–V characteristics would be possibly formed in the DEP alignment processes. [17, 18] However, the direction of rectification could not be expected. In our previous study [19], Si NW devices with rectifying I–V characteristics were fabricated by the direct current (DC) DEP method and asymmetric local Joule heating in the electrical measurement process. The direction of rectification could be determined by the voltage sweep direction.

This study presents an easy method of fabricating a controllable rectification direction of rectifying device that exhibits ZnO NW DEP alignment by a DC electric field. Such a device was found to have excellent properties for sensing UV light.

## Methods

First, zinc acetate dehydrate (Zn(CH_3_COO)_2_·2H_2_O) was dissolved in a mixed solution of monoethanolamine (C_2_H_7_NO) and isopropyl alcohol (C_3_H_8_O). The concentration of zinc acetate and ethanolamine was 0.75 M. The resultant solution was stirred at 60 °C for 120 min to yield a homogeneous colloid solution, which served as a coating solution. This coating colloid solution (40 μL) was dropped on 1 × 1 cm^2^ Si substrates for spin coating. The substrates were dried at 100 °C for 30 min and then annealed at 300 °C for 30 min. The resulting substrates are referred to as “pre-treated substrates” below.

ZnO growth solutions were prepared by mixing zinc acetate (0.04 M) with hexamethylenetetramine (HMTA) (0.04 M) while their volume ratio was maintained at 1:1. The pre-treated substrates were immersed in the ZnO growth solution (150 ml) for 60 min at 90 °C. The substrates were then removed from the solution, rinsed with deionized water, and finally dried in air. The resulting substrates were Si substrates on which were ZnO NW arrays.

**Fig. 1:**
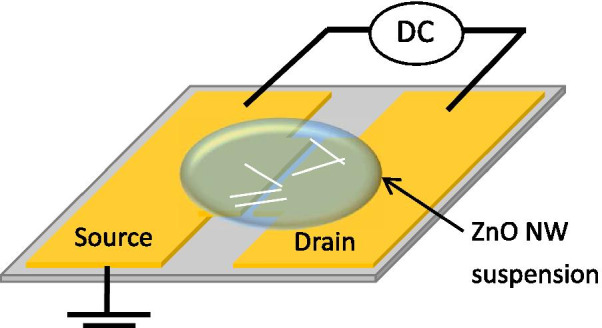
Schematics of the ZnO NWs alignment by DC DEP across Au electrodes

Au/Ti electrodes with a spacing of 2 μm were deposited on Si substrates by e-beam evaporation. An ZnO NW array was immersed in isopropyl alcohol solution (4 ml) and ultrasonicated for 15 min. The ZnO NWs fell from the Si substrate and dispersed in the solution. A droplet of ZnO NW suspension with a particular concentration was dripped over the electrode system, and then, a DC electric field was applied to the electrode pair using a power meter (Keithley, 2612A). Drain and source were connected to the positive voltage and ground, respectively. Figure 1 shows the experimental setup of the electrode system.

The surface morphology of the samples was studied by field emission scanning electron microscopy (FESEM, JEOL, JSM-6700F). Atomic images of nanowires were obtained using a high-resolution transmission electron microscope (HRTEM, JOEL, JEM-2100F). The crystal structure of the nanowires was characterized by X-ray diffraction (XRD, Mac Science, MXP-III).

## Results and Discussion

**Fig. 2:**
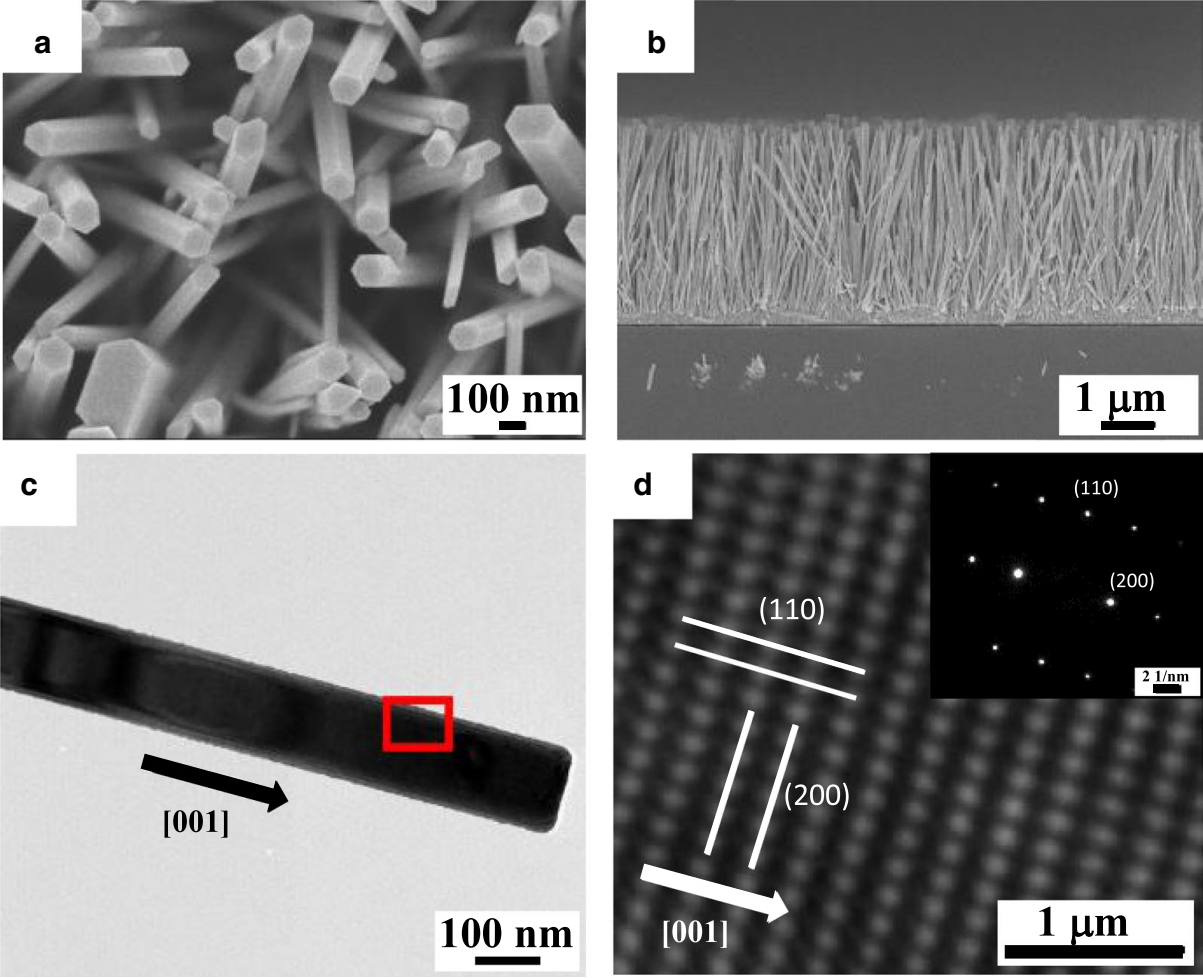
**a** Top-view and **b** cross-sectional SEM images of ZnO NWs arrays fabricated by DC-DEP method. **c** TEM image of ZnO NW. **d** Atomic resolution TEM image of ZnO NW corresponds to the red square in **c**. The inset is the SAD patterns of ZnO NW

Figure 2a, b shows the plan view and cross-sectional SEM images, respectively, of ZnO NW arrays that were grown by the hydrothermal method. The ZnO NWs had a hexagonal shape, a mean diameter of 120 nm, and a length of 3.5 μm. Figure 2c shows the TEM image of an individual ZnO NW, which is a single-crystalline structure and the growth direction of [001], as confirmed by the atomic resolution TEM image in Fig. 2d.

**Fig. 3:**
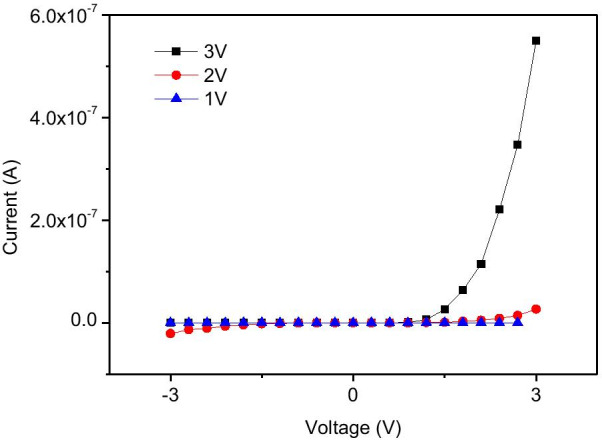
I–V curves of the individual ZnO NW device with voltages of 1, 2, and 3 V applied to the drain electrode in the DEP alignment process

The concentration of the original ZnO NW suspension was estimated to be about 2.5 × 10^6^ #/μl. The original ZnO NW suspension was diluted by 20 × and dropped onto the separated Au/Ti electrodes with a DC bias of 1 to 7 V in the DEP alignment process. The ZnO NWs aligned across the Au/Ti electrodes, parallel to each other at a voltage from 1 to 3 V; the density of the aligned ZnO NWs increased with the applied bias (Additional file 1: Fig. S1). However, when the applied bias exceeded 4 V the electrodes broken easily (Additional file 1: Fig. S2). The density of the aligned ZnO NWs was controlled by varying the concentration of the ZnO NW suspension. Therefore, to fabricate an individual ZnO NW device for the purposes of measuring its electrical property, different concentrations of ZnO NW suspensions were used at applied voltages of 1, 2, and 3 V. Figure 3 plots the I–V curves of the fabricated individual ZnO NW devices with voltages of 1, 2, and 3 V applied to the drain electrode in the DEP alignment process. Rectifying behavior was observed when the applied voltage was 3 V. The I–V curves of about 70% of the devices exhibited rectifying behavior and those of other devices had contact resistance. When the applied voltage was smaller than 2 V almost all devices had high contact resistance.

**Fig. 4:**
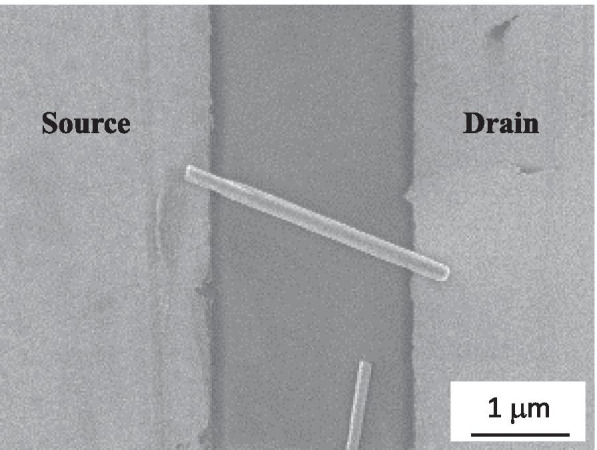
SEM image of the individual ZnO NW device that was fabricated using a voltage of 3 V in the alignment process and exhibited a rectifying IV characteristic

**Fig. 5:**
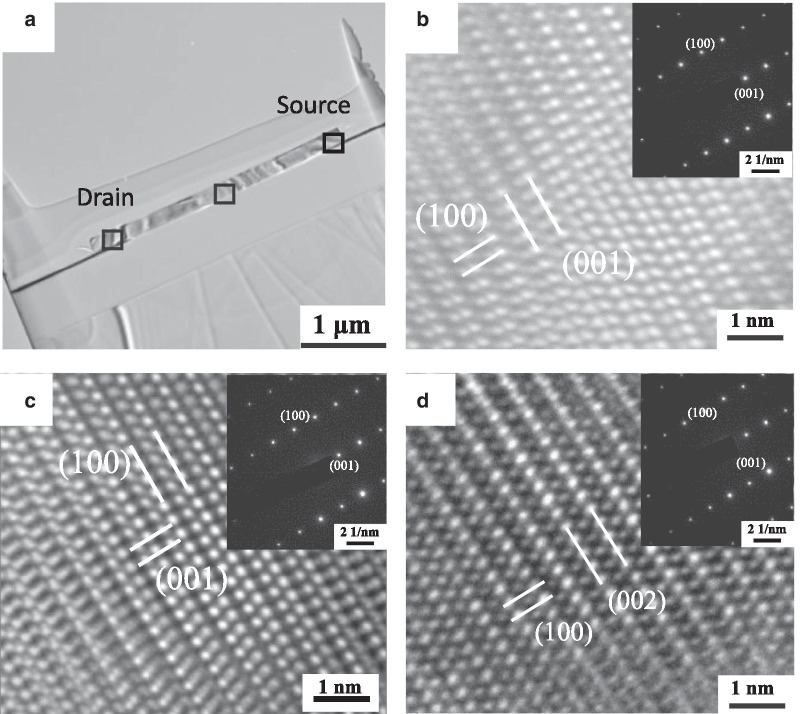
**a** TEM images of the individual ZnO NW device that was fabricated using a voltage of 3 V in the alignment process and exhibited a rectifying IV characteristic. The atomic TEM image of the drain side of ZnO NW and the area corresponds to the left square in **a**. The atomic TEM image of the middle of ZnO NW and the area corresponds to the middle square in **a**. The atomic TEM image of the source side of ZnO NW and the area corresponds to the right square in **a**

**Fig. 6:**
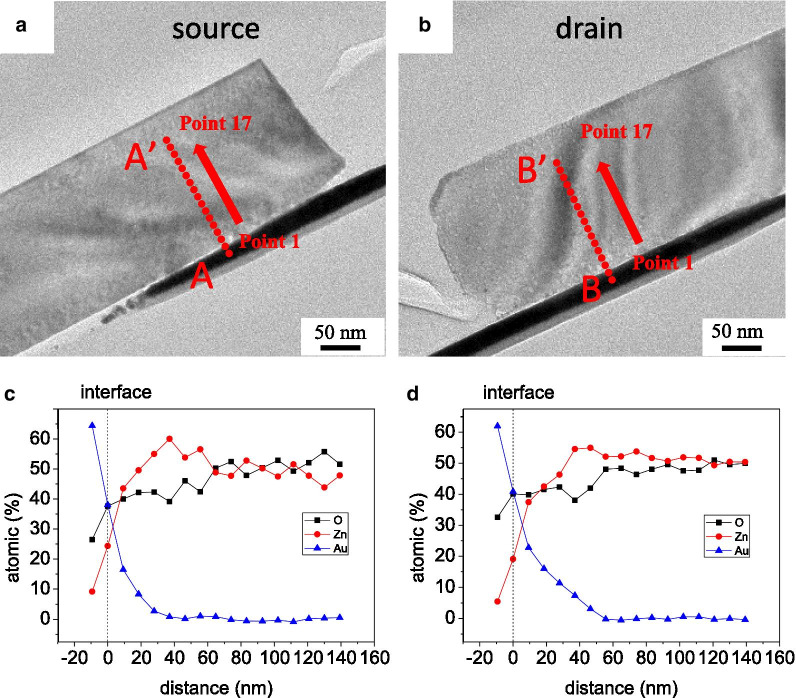
Chemical composition of the ZnO/Au interfaces at both sides of the ZnO NW was determined by energy-dispersive X-ray spectroscopy (EDS). The TEM images of the **a** drain and **b** source sides of ZnO NW. **c** The concentration distance profiles of Zn, O and Au along the **c** AA′ and **d** BB′ are shown in **a**, **b**, respectively

Figure 4 shows the SEM image of the individual ZnO NW device that was fabricated using a voltage of 3 V in the alignment process and exhibited a rectifying IV characteristic. The TEM images and select area electron diffraction (SAED) analysis of this rectifying device are shown in Fig. 5. The crystal structures at the source and drain sides and middle of the nanowire were all the same as that of the ZnO NW before alignment, implying that the alignment process does no significant structural damage to nano-objects. In order to understand why the I–V curve exhibited rectifying behavior, the chemical composition of the ZnO/Au interfaces at both ends of the ZnO NW was determined by energy-dispersive X-ray spectroscopy (EDS), as shown in Fig. 6. The concentration-distance profile of Au implies that Au diffused from the electrode to ZnO NW. Both atomic concentrations of Zn and O about 60–140 nm away from the ZnO/Au interface were about 50%. Toward the interface, the concentration of Zn first increased slightly and then decreased rapidly, while the concentration of O decreased slowly. We infer the following reasons. The ZnO/Au interface exhibited contact resistances when the ZnO NW was adsorbed on both Au electrodes in the DEP alignment process. The temperature of the NW/electrode contacts increased with the high electron flux flow through the contacts due to Joule heating [20], which caused the Au atoms to diffuse from the Au electrodes to the inner ZnO NWs. Zn atoms were pushed to the inside of ZnO NW and Zn vacancies formed.

Figure 6 indicates that the Au concentration at the drain side is higher than that at the source side, indicating that the temperature at the drain side was higher than that at the source side due to the asymmetric Joule heating effect. In addition, a few devices that were fabricated with a DC bias of 3 V were deformed at the drain side, as shown in Additional file 1: Fig. S3. For the devices that were fabricated under applied bias of 5 V and 7 V DC, the anode regions were severely damaged by melting then the cathode regions, as shown in Additional file 1: Fig. S2. These phenomena also indicate that the Joule heating was asymmetric.

**Fig. 7:**
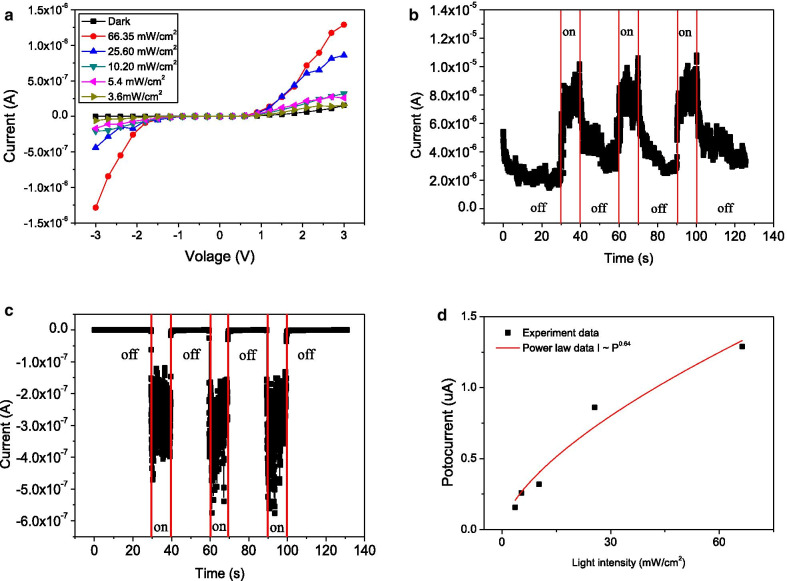
**a** I–V curves of a ZnO-NW-based sensor under 365 nm light irradiation of different intensities. **b** Time-resolved photoresponse of a ZnO-NWs-based sensor at + 3 V in reverse-biased mode under white UV illumination by switching on and off. **c** Time-resolved photoresponse of a ZnO-NWs-based sensor at − 3 V in reverse-biased mode under white UV illumination by switching on and off. **d** Photocurrent of a ZnO-NW-based sensor at − 3 V in reverse-biased mode as a function of light intensity and corresponding fitting curve using the power law

To investigate the photosensing properties of the rectifying ZnO NW-based device, 365 nm UV light with various intensities were vertically shone onto the devices while the corresponding photoresponse characteristics were recorded. Figure 7a plots the I–V curves of this device, which reveals that the photocurrent could be induced. Figure 7b, c shows the time-dependent photoresponse of this rectifying device under periodic illuminations. A higher sensitivity was achieved when the device exhibited reverse bias. The photosensitivity (S) was calculated using following equation [21],

$$S = \frac{{I_{{\text{UV/vis}}} }}{{I_{{{\text{dark}}}} }}$$

where *I*_UV/Vis_ and *I*_dark_ are the currents that were measured under illumination and in the dark, respectively. The response time and recovery time are defined as the times required for the sensor to reach 90% of its steady resistance and back to 10% of the value. [22] As shown in Fig. 7b, when the device was under UV excitation at + 3 V in the forward-biased mode, the current increased from ~ 2.5 to ~ 5.75 μA. The sensitivity was 2.3 and response and recovery times were 1.8 s and 4.9 s, respectively. On the other hand, when the device was under UV light excitation at − 3 V in reverse-biased mode, as shown in Fig. 7c, the current increased abruptly from 0.1 to 200 nA. The sensitivity was 2000, which was 870 times that of the device in forward-biased mode. The response time and recovery times were 0.1 and 0.145 s, respectively, which were much shorter than those in forward-biased mode. The Pt(Ga)-ZnO NW-Pt Schottky detector prepared by Zhou et al. [13] exhibits the sensitivity of 1500 at 1 V in reverse-biased mode under 365 nm UV radiation. The response time and recovery times were 0.6 and 6 s. Compared with their device, the device in this work has higher response and recovery speed and simpler fabrication process. Thus, this method can be considered to fabricate other Schottky diodes based on semiconductor nanowires. Figure 7d shows the photocurrent (*I*_P_) of a ZnO-NW-based sensor at − 3 V in reverse-biased mode can match a simple power law, *I*_P_ ∝ *P*^0.64^, where P is the light intensity. The non-unity exponent is a result of the complex process of electron–hole generation, trapping, and recombination within the semiconductor. [23]

**Fig. 8:**
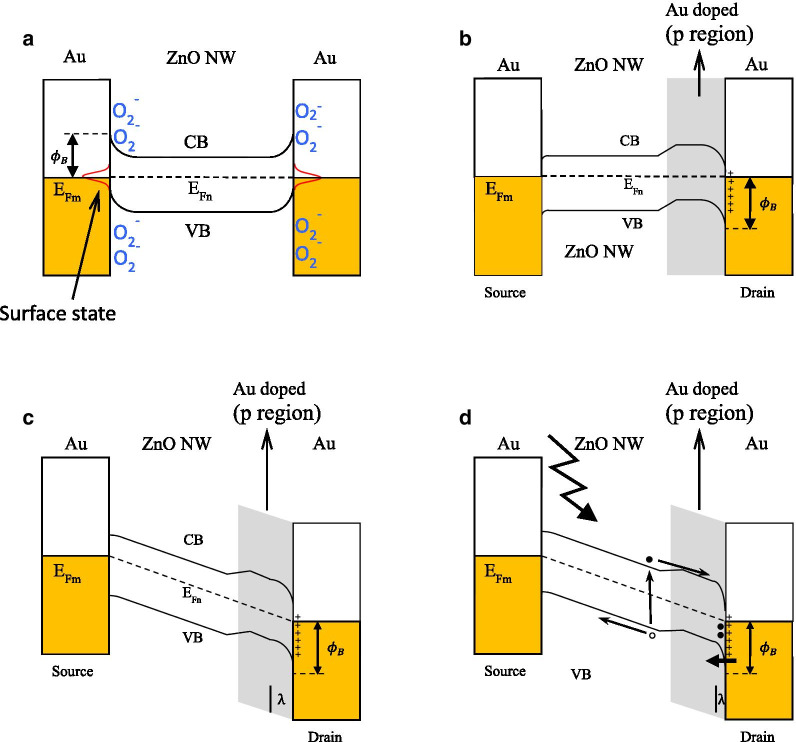
Band diagrams of Au/ZnO/Au structure. **a** The device formed by the DEP alignment process with the applied DC bias of less than 2 V. **b** The device formed by the DEP alignment process with the applied DC bias of 3 V, **c** dark conditions under reverse bias and **d** under illumination with reverse bias. *λ* is the depletion width

In this work, the devices have the metal–semiconductor–metal structure (M-S-M). Oxygen adsorbed on the ZnO surface in air and captured free electrons, which causes the depletion region near the surface. When the applied DC bias was less than 2 V in the DEP alignment process, ZnO NW just physically adsorbed on the Au electrode. The Au/Zn interface has serious contact resistance due to the formation of Schottky contacts. The energy diagram is shown in Fig. 8a. Thus, the current cannot flow through the device in I–V curve measurement as shown in Fig. 3. When the applied bias arose to 3 V, asymmetric Joule heating occurred and oxygen desorbed to form Au/ZnO interface. Simultaneously, the Au atoms diffused from the Au electrodes to the ZnO NWs and Zn vacancies generated. Previous study [24] shows that ZnO NWs fabricated by the hydrothermal method were n-type semiconductor with the work function of 5.28 eV because of O vacancies formation. In theory, the Au/ZnO interface exhibited the characteristics of Ohmic contact. When the concentration of Zn vacancies was higher than that of O vacancies, the characteristic of ZnO nanowire transformed from n-type to p-type semiconductor because Zn vacancies played an acceptor-like role. Thus, the Au/ZnO interface transformed its electric property to Schottky contact. [19] In this study, the asymmetric Joule heating caused the Schottky contact at the drain end and Ohmic contact at the source end as shown in Fig. 8b. Therefore, if the applied bias sufficed to induce Joule heating (3 V), the rectifying IV curve was obtained.

With respect to photoresponse performance, the rectifying device in reverse-biased mode had a high sensitivity and short response time. The band diagram of the device with a reveres bias in dark is shown in Fig. 8c. The large depletion region (*λ*) hinders the carrier flow and reduces the dark current. UV illumination, the band diagram is shown in Fig. 8d. The photogenerated electrons created in the depletion region of the reverse-biased Schottky junction are trapped in the depletion regions, which shrink the depletion region. The accumulated electron will attract holes from the electrode into the nanowire. The narrowing of depletion region causes the holes tunneling in the semiconductor, causing the enhancement of a current gain greater than unity and the increase of the hole transport speed [23, 25, 26]. In additional, the pn junction in the ZnO nanowire forms a potential barrier when the photodetector is reverse biased. Thus, the I–V curves of the device under 365 nm light irradiation were not linear, as shown in Fig. 7a.

In previous studies [17, 18], rectifying devices have been fabricated by aligning ZnO NWs on paired electrodes using DEP alignment and their rectifying behavior was the result of the formation of asymmetric contacts in the DEP aligning process. However, the direction of rectification was random. In our previous study [20], the rectifying I–V characteristics of these devices was obtained in the I–V curve measurement process, and the direction of rectification was determined by the voltage sweep direction. In this study, a simple manufacturing process was used. The device was fabricated by the DC in an electric field induced assembly process and the direction of rectification was determined by the direction of current.

## Conclusions

The ZnO NW-based devices were fabricated by aligning the single-crystalized ZnO NWs across the Au electrodes using DC DEP method. The rectifying I–V characteristics of these devices can be obtained, and the direction of rectification can be determined by the direction of current due to the asymmetric Joule heating in the DEP alignment process. Joule heating caused the Au atoms to diffuse from the Au electrodes to the inner ZnO NWs and the formation of Schottky contact at the Au/ZnO interface. A fast and sensitive photoresponse was achieved for the rectifying devices in reverse-biased mode due to the carrier injection and photocurrent gain under UV illumination. This rectifying ZnO NW-based devices have potential use as photodetectors and other applications such as logic gates or sensors.

## Supplementary Information

**Additional file 1**.** Fig. S1**. SEM images of the ZnO NWs aligned across the Au/Ti electrodes with a DC bias of (a) 1 V, (b) 2 V and (c) 3V, in the dielectrophoresis alignment process.** Fig. S2**. SEM images of ZnO NW-based devices after 5 V and 7 V were applied to the drain electrode for ZnO NW alignment.** Fig. S3**. The SEM image of the individual ZnO NW device that was fabricated using a voltage of 3 V in the alignment process and was deformed at the drain side.

## Data Availability

The datasets used and/or analyzed during the current study are available from the corresponding author on reasonable request.

## Abbreviations

ZnO: Zinc oxide
Au: Gold
NWs: Nanowires
UV: Ultraviolet
IV: Current voltage
Cu: Copper
Pt: Platinum
Ga: Gallium
FIB: Focus ion beam
AC: Alternating current
DEP: Dielectrophoresis
DC: Direct current
HMTA: Hexamethylenetetramine
TEM: Transmission electron microscope
SADE: Select area electron diffraction
EDS: Energy-dispersive X-ray spectrometer
O: Oxygen
