# Supplementary material for: Fabrication of Rectification Nanosensors by Direct Current Dielectrophoresis Alignment of ZnO Nanowires
Source: Nanoscale Res Lett. 2021 May 19;16:86. doi: 10.1186/s11671-021-03539-6 (PMC8134615; doi:10.1186/s11671-021-03539-6)
Supplement: Supplementary file 1 — Additional file 1. Fig. S1. SEM images of the ZnO NWs aligned across the Au/Ti electrodes with a DC bias of (a) 1 V, (b) 2 V and (c) 3V, in the dielectrophoresis alignment process. Fig. S2. SEM images of ZnO NW-based devices after 5 V and 7 V were applied to the drain electrode for ZnO NW alignment. Fig. S3. The SEM image of the individual ZnO NW device that was fabricated using a voltage of 3 V in the alignment process and was deformed at the drain side. [file 11671_2021_3539_MOESM1_ESM.pdf]

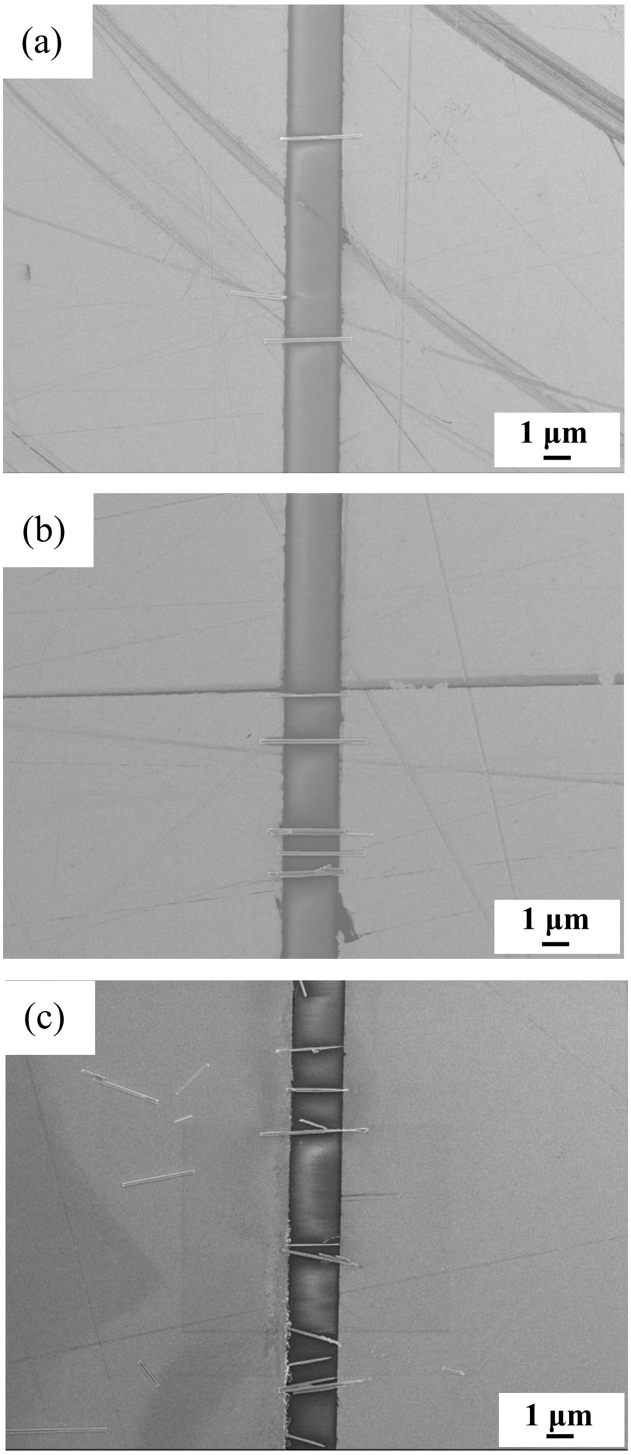

Fig. S1 SEM images of the ZnO NWs aligned across the Au/Ti electrodes with a DC bias of (a) 1 V, (b) 2 V and (c) 3V, in the dielectrophoresis alignment process

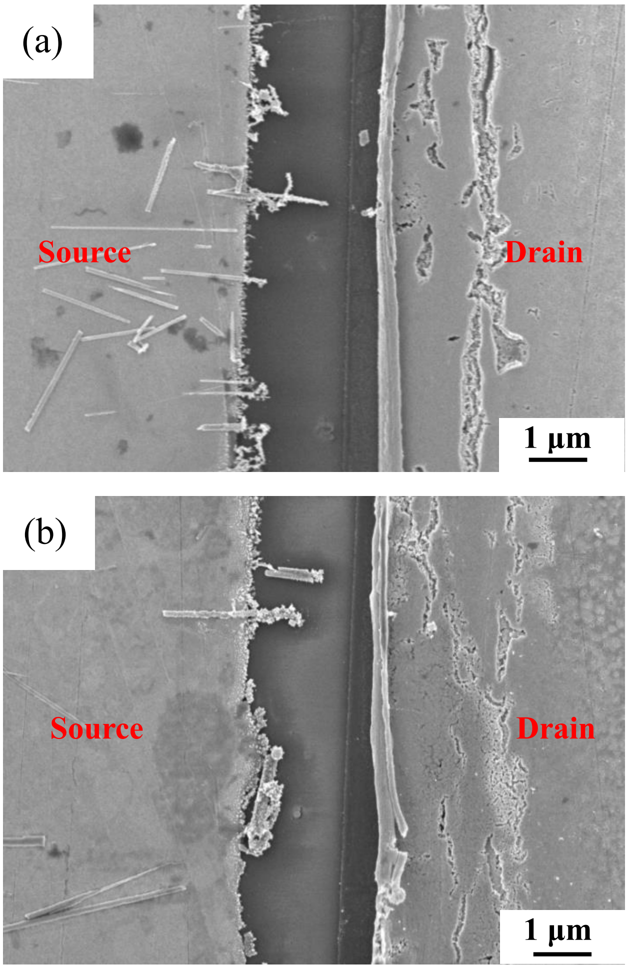

Fig. S2 SEM images of ZnO NW-based device after 5 V and 7 V were applied to the drain electrode for ZnO NW alignment.

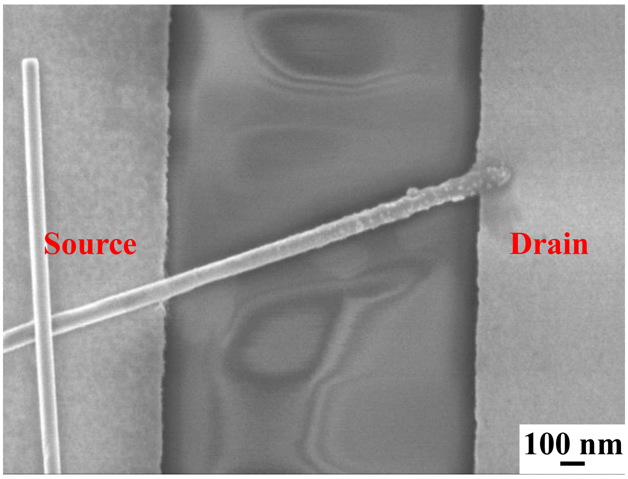

Fig. S3 The SEM image of the individual ZnO NW device that was fabricated using a voltage of 3 V in the alignment process and was deformed at the drain side.
